# Syntheses, Structures, and Characteristics of Three Metal Complexes Constructed Using Hexacarboxylic Acid

**DOI:** 10.3390/molecules24244431

**Published:** 2019-12-04

**Authors:** Lingshu Meng, Lun Zhao, Guanlin Guo, Xin Liu, Zhijun Liang, Jian Xiu, Xu Zhou

**Affiliations:** College of Chemistry, Changchun Normal University, Changchun 130032, China

**Keywords:** coordination polymers, hexacarboxylic acid, fluorescent probe, electrochemical behaviors

## Abstract

In this study, three new 3D coordination polymers (CPs), {[Cd_3_(L)(H_2_O)_6_]·H_2_O}_n_ (**1**), {[Cu_1.5_(L)_0.5_(bimb)_1.5_]·5H_2_O·DMF}_n_ (**2**), and {[Mn_1.5_(H_3_L)(bibp)_0.5_(H_2_O)_2_]·3H_2_O}_n_ (**3**) (*bimb*= 1,3-bis(imidazol-1-yl)benzene, *bibp*= 1,4-bis((4-imidazol-1-yl)benzyl)piperazine), were prepared under solvothermal or hydrothermal conditions based on a hexadentate ligand (1,3,5-triazine-2,4,6-triamine hexa-acetic acid (H_6_L)). Structural elucidations were carried out by IR spectra along with single-crystal X-ray diffraction analysis, while thermogravimetric analysis (TGA) (dynamic and isothermal) and XRD techniques were used for property evaluations of the polymers. Furthermore, the fluorescence properties and detection of the Fe^3+^ ions in **1** were tested at room temperature, and the electrochemical behavior of **2** is also stated in this article.

## 1. Introduction

With special pore structures and potential application values, coordination polymers (CPs) over the past few decades have attracted researchers’ interest [1,2,3,4,5,6,7,8,9,10,11,12,13,14,15]. Recently, researchers have found some specially structured CPs that possess characteristic high-intensity fluorescence shooting, which could be applied to pollution object detection by contrasting fluorescent signals in different states. The detection method had high sensitivity, a fast detection speed, and the advantages of easy operation. Currently, the literature reports on the benefits of using this property to detect metal ions, aromatic nitro compounds, and other small molecules [16,17,18,19,20,21,22].

It is well known that Fe^3+^ is an indispensable trace element in the human body, playing a vital role in oxide reductase catalysis, DNA synthesis, and oxygen transport and storage [23,24,25,26,27,28,29]. A lack of Fe^3+^ might be because of anemia or heart failure, and an excess of it may damage the liver. These diseases are all serious; however, it has been reported that some CPs can selectively perceive Fe^3+^ [30,31,32]. For now, it is still necessary to synthesize CPs with higher selectivity and sensitivity, so much work needs to be done.

Furthermore, with the development of the global economy and the rapid growth of the world’s population, the demand for energy is also growing. Electric energy is one of the most important energy sources in people’s lives: Co^2+^, Cu^2+^, and Ni^2+^ plasmas have good redox activity as the central metal ions of complexes. At the same time, bulk-modified carbon paste electrodes do not dissolve in water and common organic solvents, so they are suitable electrodes for research.

**Scheme 1:**
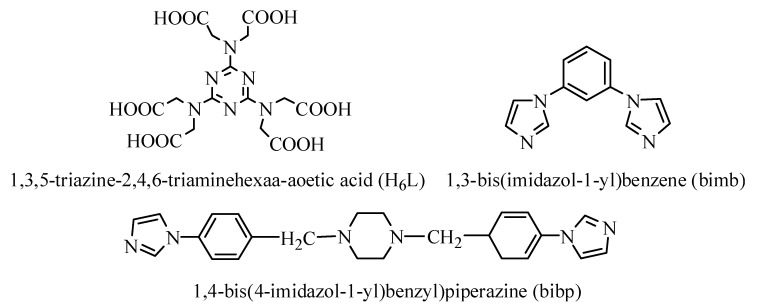
Molecular structure of several ligands.

Because of the diversity of coordination, hexacarboxylic acid could react with transition metal ions and nitrogen-containing ligands to form a variety of spatial topologies. Li and colleagues designed and synthesized two new CPs using 1,3,5-triazine-2,4,6-triamine hexa-acetic acid, and both coordination polymers had very high sensitivity to sensing properties at very low concentrations of nitro derivatives [33]. Song and coworkers synthesized three new coordination polymers with H_6_L and different ligands and indicated that these compounds displayed high-sensitivity luminescent sensing functions for nitrobenzene. Additionally, surface photovoltage spectroscopy and electric-field-induced surface photovoltage spectroscopy showed that these compounds could behave as *p*-type semiconductors [34]. Cd complexes have good luminescent properties, and they are widely used in fluorescence sensing [35]. Cu and Mn complexes have been widely reported in the fields of catalysis, magnetism, and electrochemistry [36,37]. In this work, three different metal ion complexes were synthesized under hydrothermal/solvothermal conditions using hexacarboxylic acid and two kinds of ligands (Scheme 1): they were named {[Cd_3_(L)(H_2_O)_6_]·H_2_O}_n_ (**1**), {[Cu_1.5_(L)_0.5_(bimb)_1.5_]·5H_2_O·DMF}_n_ (**2**), and {[Mn_1.5_(H_3_L)(bibp)_0.5_(H_2_O)_2_]·3H_2_O}_n_ (**3**). They were fully characterized by thermogravimetric analysis (TGA), IR spectra, photoluminescence, elemental analysis, and single-crystal X-ray diffraction analysis. These three complexes exhibited good 3D structure, and fluorescence titrations confirmed that **1** showed excellent potential in sensing Fe^3+^ in water. Moreover, the electrochemical properties of **2** were investigated by means of cyclic voltammetry.

## 2. Experimental Section

### 2.1. Materials and Methods

All solvents and reagents for synthesis were of reagent grade quality, were bought from commercial sources, and were used as received. The ligands H_6_L, bibp, and bimb were purchased from Jinan Henghua Sci. & Tec. Co. Ltd. (in Shandong Province, China). Powder X-ray diffraction (PXRD) patterns were collected on a D2 PHASER A26-X1 XRD diffractometer. IR spectra (4000–400 cm^−1^) were obtained from KBr pellets with an FTIR Nexus spectrophotometer. Elemental analyses were performed on a Perkin-Elmer 240 C analyzer. Thermogravimetric analysis (TGA) curves were measured under an air atmosphere at a heating rate of 10 °C/ min on a Perkin-Elmer TG-7 thermal analyzer. The fluorescence spectra were carried out on a HITACHI F-7000 Spectrometer. Electrochemical properties were studied on a DF-2002 electrochemical workstation [38].

### 2.2. Synthesis of {[Cd_3_(L)(H_2_O)_6_]·H_2_O}_n_ (**1**)

A mixture of Cd(NO_3_)_2_·4H_2_O (30.8 mg, 0.1 mmol), 4-(imidazol-1-ylmethyl)benzonitrile (18.3 mg, 0.1 mmol)(auxiliary agent), and H_6_L (47.4 mg, 0.1 mmol) were dissolved in H_2_O solvent (10 mL). After being mixed evenly, it was placed in a Parr Teflon-lined stainless steel vessel (20 mL) and heated at 160 °C for 72 h under autogenous pressure. Colorless crystals were acquired. The reaction yield was ca. 60% based on an H_6_L ligand. The elemental analysis for C_15_H_26_Cd_3_N_6_O_19_(%): C, 19.34; H, 2.81; N, 9.02. Found: C, 19.71; H, 2.89; N, 8.86. FT-IR (4000–400 cm^−1^): 3214 (m), 1570 (w), 1483 (w), 1440 (w), 1197 (m), 1180 (s), 986 (s), 818 (s), 719 (m).

### 2.3. Synthesis of {[Cu_1.5_(L)_0.5_(bimb)_1.5_]·5H_2_O·DMF}_n_ (**2**)

A mixture of Cu(NO_3_)_2_·3H_2_O (24.2 mg, 0.1 mmol), bimb ligand (21.1 mg, 0.1 mmol), and H_6_L (47.4 mg, 0.1 mmol) were dissolved in DMF/H_2_O/0.1MHNO_3_ solvent (8:2:1, 11 mL). After being mixed evenly, it was placed in a Parr Teflon-lined stainless steel vessel (20 mL) and heated at 80 °C for 72 h under autogenous pressure. Aquamarine crystals were acquired. The reaction yield was ca. 47% based on an H_6_L ligand. The elemental analysis for C_57_H_64_N_20_Cu_3_O_24_(%): C, 42.68; H, 4.02; N, 17.47. Found: C, 42.99; H, 4.12; N, 16.68. FT-IR (4000–400 cm^−1^): 3736 (s), 3393 (s), 3130 (s), 1609 (m), 1517 (m), 1289 (m), 1250 (m), 1194 (s), 1113 (s), 988 (s), 811 (s), 792 (s).

### 2.4. Synthesis of {[Mn_1.5_(H_3_L)(bibp)_0.5_(H_2_O)_2_]·3H_2_O}_n_ (**3**)

A mixture of MnCl_2_·4H_2_O (19.7 mg, 0.1 mmol), bibp ligand (39.8 mg, 0.1 mmol), and H_6_L (47.4 mg, 0.1 mmol) were dissolved in DMF/H_2_O/0.1MHNO_3_ solvent (8:2:1, 11 mL). After being mixed evenly, it was placed in a Parr Teflon-lined stainless steel vessel (20 mL) and heated at 80 °C for 72 h under autogenous pressure. Brown crystals were acquired. The reaction yield was ca. 51% based on an H_6_L ligand. The elemental analysis for C_54_H_50_Mn_3_N_18_O_34_(%): C, 39.12; H, 3.01; N, 15.22. Found: C, 39.07; H, 3.04; N, 15.19. FT-IR (4000–400 cm^−1^): 3414 (s), 1552 (m), 1481 (s), 1315 (s), 1200 (m), 1062 (s), 988 (s), 855 (s), 809 (m).

### 2.5. X-ray Crystallography

Appropriate single crystals of compounds **1**–**3** were mounted on a glass fiber, and the intensity data were measured at 293 K on a Bruker SMART APEXⅡCCD area detector with graphite monochromatic Mo–Kα radiation using the ω scan mode (λ = 0.71073 Å). Data reduction and cell refinement were performed with SADABS software packages. Absorption corrections were done using the empirical method, and all of the structures were solved with the direct method using SHELXS-97 [39]. The anisotropy of all nonhydrogen atoms determined their coordinates. The crystallographic data inferred and other relevant information about structural determination are shown in Table 1.

## 3. Results and Discussion

### 3.1. Structural Description of **1**

**Scheme 2:**
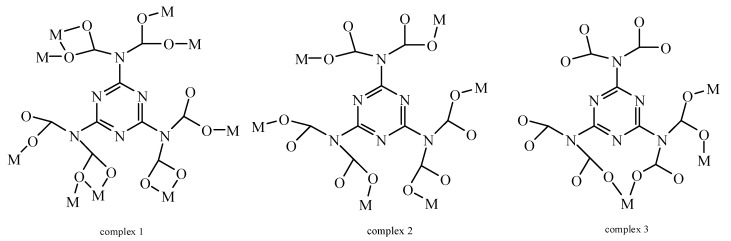
Coordination mode of carboxylic acid.

**Figure 1:**
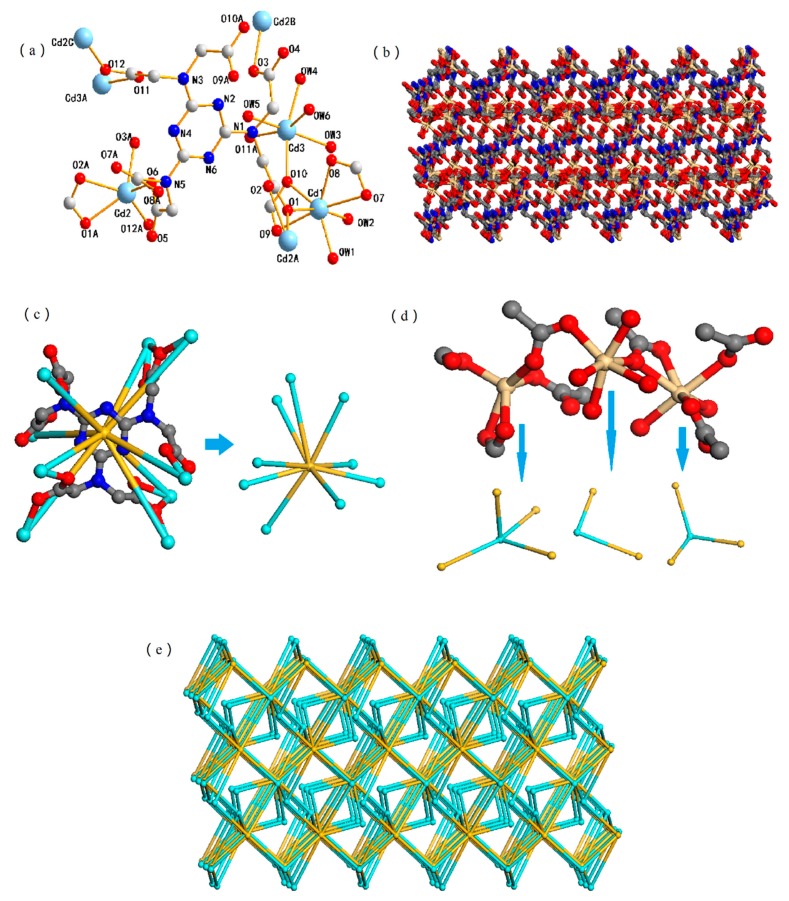
(**a**) The coordination environment for the three Cd(II) ions in **1**. (**b**) 3D framework with L^6−^ and Cd(II) ions. (**c**) Nine-coordinate node of carboxylic acid L^6−^. (**d**) Three-coordinate node and four-coordinate node of Cd(II) ions. (**e**) 3D network topology representation of **1**.

CP **1** was crystallized in a monoclinic space group, *P2*_1_/*c*, and it contained one carboxylic acid L^6−^ ligand, three crystallographically independent Cd(II) ions, six coordinated H_2_O molecules, and one free H_2_O molecule (Figure 1a). Each Cd(II) ion (Cd1, Cd2, and Cd3) had a distinct coordination environment. In detail, Cd 1 constituted a deformed octahedron with seven coordinates (coordination of Cd 1 with O1, O7, O8, O9, O10, OW1, and OW2); Cd 2 had a five-coordinate mode (coordination of Cd 2 with O1A, O2A, O3A, O6, and O12A); and Cd 3 had a six-coordinate mode (coordination of Cd 3 with O10, O11A, OW3, OW4, OW5, and OW6). In asymmetrical structural units, the six carboxylate groups of the ligand L^6−^ exhibited four different bonding modes (Scheme 2). The distance of the Cd–O bond ranged from 2.186(2) to 2.537(2) Å. Adjacent Cd(II) ions were joined by carboxylic acid ligands L^6−^ to form a 3D framework (Figure 1b). Using a topological approach, the carboxylic acid ligand L^6−^ could be regarded as a 9-c node (Figure 1c) and the Cd(II) ion as a 3-c node or 4-c node (Figure 1d); therefore, the entire structure could be denoted by its topology (Figure 1e).

### 3.2. Structural Description of **2**

**Figure 2:**
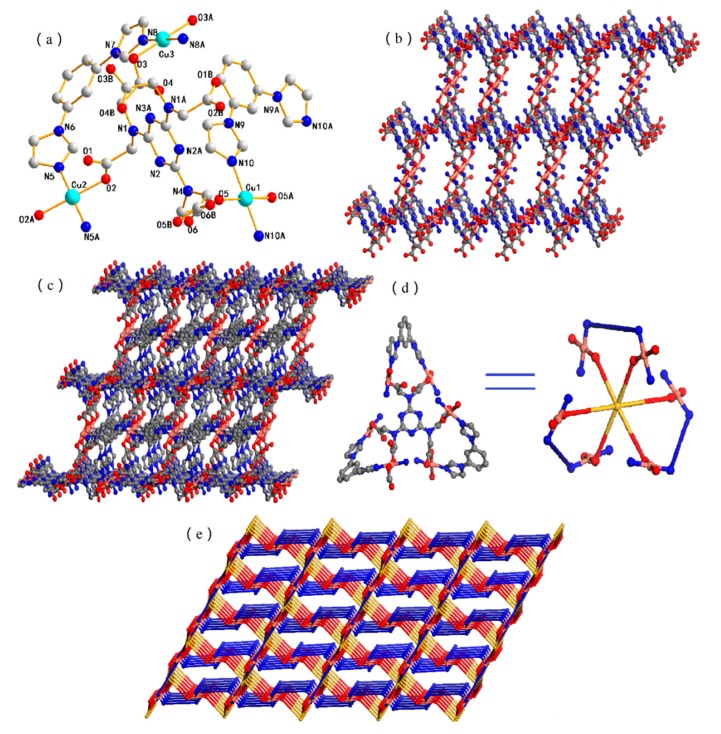
(**a**) The coordination environment for the three Cu(II) ions in **2**. (**b**) View of the 3D frame structure with L^6−^ and Cu(II) ions. (**c**) View of the 3D frame structure after it was filled with nitrogen-containing ligands. (**d**) View of a six-coordinate node and a four-coordinate node. (**e**) 3D network topology representation of **2**.

CP **2** was crystallized in a monoclinic space group, *C2/c*, and it contained 3/2 bimb ligands, a 1/2 carboxylic acid L^6−^ ligand, 3/2 crystallographically independent Cu(II) ions, five uncoordinated H_2_O molecules, and one free DMF molecule (Figure 2a). In asymmetrical structural units, the Cu(II) ions were four-connected by two oxygen atoms of L^6−^ ligands and two N atoms of bimb ligands, showing a distorted tetrahedron. Six carboxylate groups of the L^6−^ ligands exhibited a monodentate character (Scheme 2). The distance of the Cu–O bond ranged from 1.939(3) to 2.026(4) Å, and the Cu–N bond ranged from 1.975(5) to 2.021(5) Å. The Cu(II) ion was bonded to the carboxylic acid ligand L^6−^ to form a 3D framework structure (Figure 2b), and the bimb ligand was relinked to two carboxylic acid ligands of the L^6−^ ligand (Figure 2d) and populated into this 3D framework (Figure 2c). Using a topological approach, the carboxylic acid ligand L^6−^ and the Cu(II) ion could be regarded as a 6-c node and a 4-c node, respectively, and the ligand bimb could be regarded as a linker; therefore, the entire structure could be denoted by its topology (Figure 2e).

### 3.3. Structural Description of **3**

**Figure 3:**
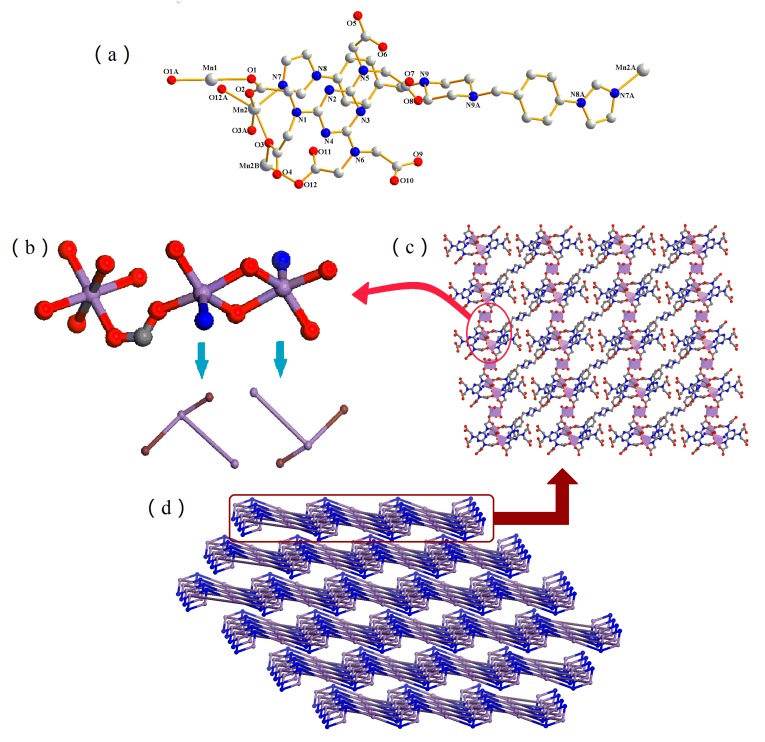
(**a**) The coordination environment for the three Mn(II) ions in **3**. (**b**) View of the Mn(II) ion coordination mode. (**c**) View of the 2D frame structure. (**d**) 3D network topology representation of **3**.

CP **3** was crystallized in a triclinic space group *P-1*, and it contained 1/2 bibp ligands, one H_3_L^3−^ ligand, 3/2 crystallographically independent Mn(II) ions, and five H_2_O molecules (three uncoordinated and two coordinated) (Figure 3a). In asymmetrical structural units, two Co(II) ions had different coordination environments; Mn1 was six-connected by two oxygen atoms of H_3_L^3−^ ligands and four oxygen atoms of bound water; Mn2 was five-connected by four carboxylic oxygen atoms of H_3_L^3−^ ligands and one N atom of bibp ligands; and two symmetrical Mn2 atoms formed a metal cluster, as shown in Figure 3b. Three carboxylate groups of the H_3_L^3−^ ligands exhibited a monodentate character, leaving three uncoordinated (Scheme 2). The distance of the Mn–O bond ranged from 2.107(3) to 2.232(3) Å, and the Mn–N bond length was 2.145(3) Å. In this complex, the Mn(II) ion and H_3_L^3−^ ligands formed 1D chains, and then the 1D chains formed into a 2D layer through bibp ligands (Figure 3b,c). The 3D frame was obtained by stacking the 2D layers. Using a topological approach, the Mn(II) ion (as a 3-c node) and the carboxylic acid ligand H_3_L^3−^ could be regarded as a 3-c node; therefore, the entire structure could be denoted by its topology (Figure 3d).

### 3.4. PXRD and Thermogravimetric Analysis (TGA)

The PXRD patterns for CPs **1**, **2**, and **3** were presented in Figures S1–S3 (in the Supplementary Information), respectively. The experimental data were in good agreement with the main peak of the corresponding simulations, proving their good phase purity.

TGA experiments were performed from 20 to 800 °C at a heating rate of 10 °C/min under an air atmosphere to evaluate the thermal stabilities of three complexes (Figure S4, in the ESI†). For 1, the first phase of weight loss from 25 to 350 °C was caused by a release of H_2_O molecules. Then, it continuously lost weight between 350 and 610 °C due to a collapse of the frameworks. The framework was stable up to ca. 610 °C. The final residue was CdO (obsd 45.23%, calcd 42.00%). For **2**, the first phase of weight loss from 25 to 160 °C was caused by a release of H_2_O and DMF molecules (obsd 11.11%, calcd 10.16%). Then, it continuously lost weight between 160 and 450 °C, probably due to the thermal decomposition of ligand bimb and the collapse of the frameworks. The framework was stable up to ca. 450 °C. The final residue was CuO (obsd 15.01%, calcd 14.88%). For 3, the first phase of weight loss from 25 to 105 °C was caused by a release of H_2_O molecules (obsd 8.78%, calcd 5.42%), and the framework began to decompose between 240 and 430 °C. The final residue was MnO (obsd 13.49%, calcd 12.82%).

### 3.5. Photoluminescent Properties of **1**

**Figure 4:**
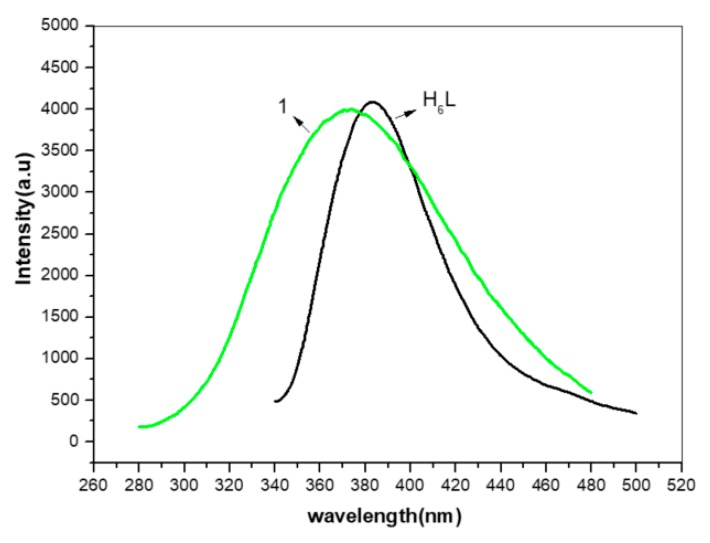
Solid-state emission spectra of **1**: H_6_L at room temperature.

The solid complex **1** and the ligand were subjected to a fluorescence test at room temperature, and the resulting spectrum is shown in Figure 4. At a maximum emission wavelength of 300 nm, **1** had an emission peak at 374 nm, and the maximum emission peak of the hexacarboxylic acid H_6_L ligand was at 383 nm at an excitation wavelength of 321 nm. Since the central metals of **1** were Cd(II) ions, which are not easily oxidized or reduced in the skeleton due to the outermost electron arrangement, there was a stable d^10^ configuration [40,41]. For complex **1**, the emissions could be essentially ascribed to the luminescence of the carboxylic acid ligand H_6_L, since a similar emission was observed in the free H_6_L ligand. The fluorescence emission peak of **1** at 374 nm (λ*ex* = 300 nm) produced a small blue shift at 383 nm (λ*ex* = 321 nm) relative to the ligand H_6_L, which was mainly due to the coordination of the ligands with the metals.

### 3.6. Detection of Fe^3+^ Ions

**Figure 5:**
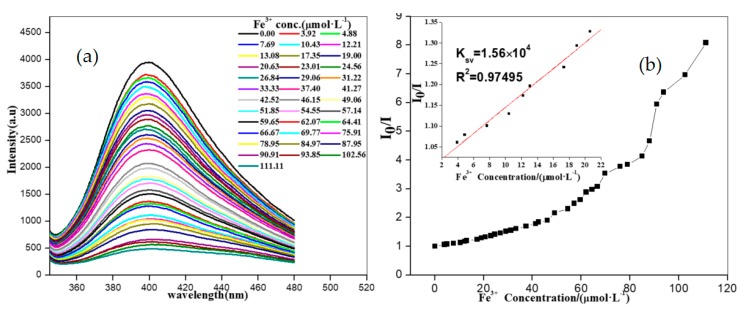
Stern–Volmer plot of **1** with 0.2 mM Fe^3+^ solution (**a**) and (**b**).

Through the literature, we discovered a fluorescence sensing experiment of Fe^3+^ with good effects on quenching. First, 2 mL of aqueous solvent suspensions containing the crystal samples of **1** (2 mg) were subjected to an ultrasound for half an hour. Next, Fe(NO_3_)_3_ (0.2 mmol·L^−1^(mM)) was gradually added, and the luminescence intensity was tested. As is depicted in Figure 5a, the fluorescence intensities of the Fe^3+^ stable suspensions of **1** were quenched gradually, decreasing as the concentration of Fe^3+^ increased, and the quenching efficiency was 87.63% when the Fe^3+^ concentrations rose to 0.1111 mM. The quenching consequent could be rationalized by the Stern–Volmer equation quantitatively: *I*_0_/*I* = *K_sv_*[*M*] + 1 (*I*_0_ and *I* are the luminescence intensities of **1** without and with the addition of an analyte, respectively, *Ksv* is the quenching constant, and [*M*] is the molar concentration of Fe^3+^) [42]. *K_sv_* was calculated to be 1.56 × 10^4^ M^−1^ from the Stern–Volmer plots (Figure 5b), and it showed a good linear relationship when the concentration was low (0.0039–0.0206 mM). The PXRD data of complex **1** in aqueous solution and Fe^3+^ solution showed its stability in solution (Figure S2 in the ESI†). Then, the UV-Vis spectra of Fe^3+^ ions and complex **1** in aqueous solution were tested (Figure S5 in the ESI†). A wide absorption band of Fe^3+^ ions in aqueous solution in the range of 270–350 nm covered the absorption bands of complex **1** in water, indicating that there was an energy competition between the Fe^3+^ ions and the complex, which ultimately led to fluorescence quenching of the complex, as has been reported in other MOF literature [43,44,45,46].

### 3.7. Electrochemical Properties

Subsequently, the electrochemical properties of **2** were studied. The bulk-modified carbon paste electrode (CPE) does not dissolve in water and common organic solvents, so we chose it. The **2**-modified CPE (**2**-**CPE**) was made with 2 mg graphite powder, 2 mg complex **2**, and the right amount of paraffin oil in an agate mortar for around half an hour to achieve even mixing. Then, it was added to a 3-mm inner diameter Teflon tube and connected to a copper wire to establish electrical contact. The cyclic voltammograms of the **2**-**CPE** in 0.5 mol·L^−1^ NaOH solution are shown in Figure S6 (ESI†). It can be seen clearly that in the potential range of +400 to −800, a reversible redox peak is observed for **2**-**CPE**, which could be attributed to the redox of Cu(II)/Cu(I) [47]. *E*_1/2_ = (*E_pa_* + *E_pc_*)/2 was −219.5 mV (50 mV/s) for **2**-**CPE**.

**Figure 6:**
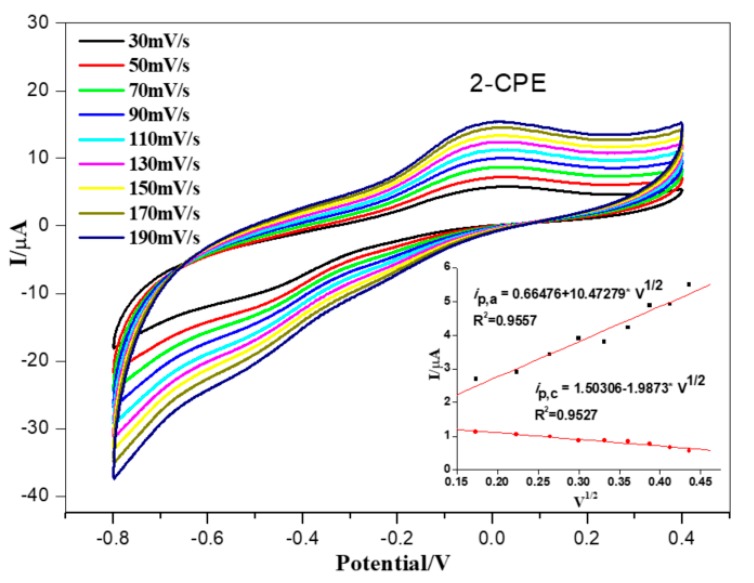
Cyclic voltammogram of **2**-**CPE** in 0.5M NaOH solution at 30 to 190 mV/s scan rates.

The scan rate’s effect on the electrochemical behavior of **2**-**CPE** was investigated in 0.5 mol·L^−1^ NaOH solution (Figure 6). At higher scan rates, the peak potentials changed gradually: the cathodic peak potentials shifted in a negative direction, and the homologous anodic peak potentials shifted in a positive direction. The inset of Figure 6 shows that the peak currents were proportional to the square root of the scan rates, suggesting that the redox processes for **2**-**CPE** are surface-controlled [48].

## 4. Conclusions

In summary, three complexes were synthesized with a hexacarboxylic acid ligand and nitrogen-containing ligands, and their PXRD, thermal stability, IR, luminescent, and electrochemical properties were also studied. In short, for **1**, adjacent Cd(II) ions were joined by carboxylic acid ligands L^6−^ to form a 3D framework. Complex **1** had better photoluminescent properties, and it could be used as a potential fluorescence material for sensing Fe^3+^ ions with high selectivity and sensitivity, where the *K_SV_* was calculated to be 1.56 × 10^4^ M^−1^. For **2**, the Cu(II) ion was bonded to the carboxylic acid ligand L^6−^ to form a 3D framework structure. For **3**, the Mn(II) ion and H_3_L^3−^ ligands formed 1D chains, and then the 1D chains constituted a 2D layer by linking with bibp ligands. Finally, a 3D frame was obtained through layering. Furthermore, **2** might have potential applications in the field of electrochemistry due to its good redox performance.

## Figures and Tables

**Table 1:** Crystal data and structure refinement for **1**, **2**, and **3.**

Compound	1	2	3
Molecular Formula	C_15_H_26_Cd_3_N_6_O_19_	C_57_H_64_N_20_Cu_3_O_24_	C_54_H_50_Mn_3_N_18_O_34_
Formula weight	917.54	1593.85	1659.94
Crystal system	Monoclinic	Monoclinic	Triclinic
Space group	*P2*_1_/*c*	*C2/c*	*P-1*
*a*/Å	9.7574 (3)	31.421 (3)	8.234 (2)
*b*/Å	15.1249 (5)	17.6474 (18)	10.610 (3)
*c*/Å	18.6267 (6)	17.2392 (17)	19.504 (6)
*α/*(°)	90	90	81.094 (6)
*β/*(°)	95.449 (1)	117.491 (2)	87.935 (7)
*γ/*(°)	90	90	86.408 (7)
*V*/nm^3^	2736.50 (15)	8479.7 (15)	1679.6 (8)
*Z*	4	4	1
*D*_c_/(g·cm^−3^)	2.227	1.248	1.641
*F*(000)	1760	3260	847
*s*	1.090	1.189	1.039
*R*_1_*/wR*_2_ [*I* > 2*σ*(*I*)]	0.0235, 0.0623	0.1020, 0.3087	0.0682, 0.1824
*R*_1_*/wR*_2_ (all data)	0.0268, 0.0640	0.1684, 0.3611	0.1115, 0.2223

## Supplementary Materials

CCDC 1896443, 1896442, and 1959345 contain the supplementary crystallographic data for complexes **1**–**3**, respectively. These data can be obtained free of charge from the Cambridge Crystallographic Data Center via www.ccdc.cam.ac.uk/data_request/cif. The following are available online at https://www.mdpi.com/1420-3049/24/24/4431/s1, Table S1: Selected Bond Lengths (Å) and Angles (deg) for compounds **1**–**3**; Figure S1: Simulated (black), experimental (red), immersed in water after 2 days (blue) and immersed in Fe^3+^ (0.01M) after 2 day (green) powder X-ray diffraction patterns of compound **1**; Figure S2: Simulated (black), experimental (red) and immersed in water after 2 days (blue) powder X-ray diffraction patterns of compound **2**; Figure S3: Simulated (black) and experimental (red) powder X-ray diffraction patterns of compound **3**; Figure S4: The TGA diagrams of **1**, **2** and **3**; Figure S5: UV-Vis spectra of Fe^3+^, complex **1** in aqueous solution; Figure S6: CV of **2**-**CPE** in 0.5 M NaOH solution at 50 mV/s.
