# Supplementary material for: Syntheses, Structures, and Characteristics of Three Metal Complexes Constructed Using Hexacarboxylic Acid
Source: Molecules. 2019 Dec 4;24(24):4431. doi: 10.3390/molecules24244431 (PMC6943621; doi:10.3390/molecules24244431)
Supplement: Supplementary file 1 [file molecules-24-04431-s001.pdf]

Syntheses, Structures, and Characteristics of Three Metal Complexes Constructed by Using Hexavalent Carboxylic Acids

Lingshu Meng, Lun Zhao*, Xin Liu, Guanlin Guo, Zhijun Liang, Jian Xiu, Xu Zhou

College of Chemistry, Changchun Normal University, Changchun, 130032, Jilin, P. R. China;
* Correspondence: E-mail: zhaolun7511@126.com; Tel.: +86-431-86168903(L Zhao)

Table S1. Diagnostic IR bandsfor Compounds **1–3**

Wave Number of Characteristic Peak（cm^{-1}）			Attribution of functional groups
(1)	(2)	(3)	
	3736		Oscillating Oscillation of O-H on Carboxyl Groups
3214	3393,3130	3414	N-H Telescopic Vibration of Nitrogen-containing Ligands
1570,1483,1440	1609,1517	1552,1481	Telescopic Vibration of Carbonyl C=O
	1289,1250	1315,1200	C-N Telescopic Vibration Absorption
1197,1180	1194,1113	1062	C-O stretching vibration of carboxylic acid
986,818,719	988,811,792	988,855,809	Out-of-plane bending vibration of aromatics C-H

Table S2. Selected Bond Lengths (Å) and Angles (deg) for Compounds **1–3**

Compound (**1**)			
Cd(1)-O(13)	2.2564(19)	Cd(2)#5-O(7)	2.457(2)
Cd(1)-O(3)#1	2.261(2)	Cd(2)#6-O(2)	2.232(2)
Cd(1)-O(14)	2.297(2)	Cd(3)#3-O(1)	2.2668(19)
Cd(1)-O(12)	2.3525(19)	Cd(2)#3-O(10)	2.255(2)
Cd(1)-O(11)	2.4285(19)	Cd(1)#7-O(4)	2.537(2)
Cd(1)-O(8)	2.4450(19)	Cd(1)#7-O(3)	2.2605(19)
Cd(1)-O(4)#1	2.537(2)	Cd(2)#5-O(8)	2.2948(19)
Cd(2)-O(5)	2.186(2)	Cd(2)-O(7)#4	2.457(2)
Cd(2)-O(2)#2	2.232(2)	O(13)-Cd(1)-O(3)#1	129.64(7)
Cd(2)-O(10)#3	2.255(2)	O(13)-Cd(1)-O(14)	83.60(9)
Cd(2)-O(8)#4	2.2948(19)	O(3)#1-Cd(1)-O(14)	107.86(10)
Cd(3)-O(1)#3	2.2668(19)	O(13)-Cd(1)-O(12)	89.96(7)
Cd(3)-O(18)	2.272(2)	O(3)#1-Cd(1)-O(12)	134.80(7)
Cd(3)-O(11)	2.2981(19)	O(14)-Cd(1)-O(12)	95.91(9)

Cd(3)-O(17)	2.302(2)	O(13)-Cd(1)-O(11)	141.38(7)
Cd(3)-O(16)	2.315(2)	O(3)#1-Cd(1)-O(11)	88.94(7)
Cd(3)-O(15)	2.353(2)	O(14)-Cd(1)-O(11)	85.99(8)
O(11)-Cd(1)-O(8)	99.80(7)	O(12)-Cd(1)-O(11)	54.30(6)
O(3)#1-Cd(1)-O(4)#1	53.96(7)	O(13)-Cd(1)-O(8)	85.29(7)
O(12)-Cd(1)-O(4)#1	170.14(7)	O(3)#1-Cd(1)-O(8)	83.02(7)
O(8)-Cd(1)-O(4)#1	99.22(8)	O(14)-Cd(1)-O(8)	167.88(9)
O(14)-Cd(1)-O(4)#1	83.65(9)	O(12)-Cd(1)-O(8)	79.24(7)
O(11)-Cd(1)-O(4)#1	135.30(7)	O(13)-Cd(1)-O(4)#1	80.20(7)
O(5)-Cd(2)-O(2)#2	114.89(9)	O(5)-Cd(2)-O(10)#3	91.04(9)
O(2)#2-Cd(2)-O(10)#3	104.03(8)	O(5)-Cd(2)-O(8)#4	126.23(9)
O(2)#2-Cd(2)-O(8)#4	90.95(7)	O(10)#3-Cd(2)-O(8)#4	129.14(7)
O(5)-Cd(2)-O(7)#4	94.66(10)	O(2)#2-Cd(2)-O(7)#4	144.87(7)
O(10)#3-Cd(2)-O(7)#4	93.45(8)	O(8)#4-Cd(2)-O(7)#4	55.02(6)
C(1)-O(2)-Cd(2)#6	133.71(17)	O(1)#3-Cd(3)-O(18)	78.63(8)
O(1)#3-Cd(3)-O(11)	96.17(7)	O(18)-Cd(3)-O(11)	93.20(9)
O(1)#3-Cd(3)-O(17)	114.71(8)	O(18)-Cd(3)-O(17)	165.17(9)
O(11)-Cd(3)-O(17)	79.38(8)	O(1)#3-Cd(3)-O(16)	98.26(9)
O(18)-Cd(3)-O(16)	98.18(10)	O(11)-Cd(3)-O(16)	163.12(9)
O(17)-Cd(3)-O(16)	86.64(9)	O(1)#3-Cd(3)-O(15)	155.62(8)
O(18)-Cd(3)-O(15)	78.46(8)	O(11)-Cd(3)-O(15)	93.30(7)
O(17)-Cd(3)-O(15)	89.09(8)	O(16)-Cd(3)-O(15)	76.93(8)
Symmetry codes: #1 -x-1,y+1/2,-z+3/2; #2 x-1,y,z; #3 -x-1,-y+1,-z+2; #4 x,-y+1/2,z+1/2; #5 x,-y+1/2,z-1/2; #6 x+1,y,z; #7 -x-1,y-1/2,-z+3/2			
Compound (**2**)			
Cu(2)-N(5)	1.990(5)	Cu(02)-N(8)	1.975(5)
Cu(1)-N(10)	2.021(5)	Cu(2)-O(2)	1.939(3)
Cu(02)-O(3)	2.026(4)	Cu(1)-O(5)	1.947(4)
Cu(02)-N(8)#3	1.975(5)	Cu(02)-O(3)#3	2.026(4)
Cu(1)-O(5)#4	1.947(4)	Cu(1)-N(10)#4	2.021(5)
Cu(2)-O(2)#5	1.939(3)	Cu(2)-N(5)#5	1.990(5)
N(8)-Cu(02)-O(3)#3	89.5(2)	N(8)-Cu(02)-N(8)#3	180.0(3)
N(8)-Cu(02)-O(3)	90.5(2)	N(8)#3-Cu(02)-O(3)#3	90.5(2)
O(3)#3-Cu(02)-O(3)	180.000(1)	N(8)#3-Cu(02)-O(3)	89.5(2)
O(5)-Cu(1)-N(10)	88.93(19)	O(5)-Cu(1)-O(5)#4	180.0(4)
O(5)-Cu(1)-N(10)#4	91.07(19)	O(5)#4-Cu(1)-N(10)	91.07(19)
N(10)-Cu(1)-N(10)#4	180.00(8)	O(5)#4-Cu(1)-N(10)#4	88.93(19)
O(2)-Cu(2)-N(5)	90.21(16)	O(2)-Cu(2)-O(2)#5	180.00(2)
O(2)-Cu(2)-N(5)#5	89.79(16)	O(2)#5-Cu(2)-N(5)	89.79(16)
N(5)-Cu(2)-N(5)#5	180.00(17)	O(2)#5-Cu(2)-N(5)#5	90.21(16)
Symmetry codes: #1 -x+2,y,-z+1/2; #2 -x+2,y,-z+3/2; #3 -x+2,-y+1,-z+1; #4 -x+2,-y,-z+1; #5			

-x+5/2,-y+1/2,-z+1			
Compound (**3**)			
Mn(2)-O(2)	2.107(3)	Mn(2)-O(3)	2.112(3)
Mn(2)-O(12)#1	2.141(3)	Mn(2)-N(7)	2.145(3)
Mn(2)-O(3)#1	2.232(3)	O(3)-Mn(2)#1	2.232(3)
Mn(1)-OW1	2.178(3)	Mn(1)-OW1#3	2.178(3)
Mn(1)-OW2	2.219(3)	Mn(1)-OW2#3	2.219(3)
Mn(1)-O(1)	2.220(3)	Mn(1)-O(1)#3	2.220(3)
O(12)-Mn(2)#1	2.141(3)		
O(2)-Mn(2)-O(3)	101.08(12)	O(2)-Mn(2)-O(12)#1	84.69(12)
O(3)-Mn(2)-O(12)#1	144.49(11)	O(2)-Mn(2)-N(7)	100.20(13)
O(3)-Mn(2)-N(7)	109.62(12)	O(12)#1-Mn(2)-N(7)	103.62(12)
O(2)-Mn(2)-O(3)#1	165.34(11)	O(3)-Mn(2)-O(3)#1	74.51(12)
O(12)#1-Mn(2)-O(3)#1	91.07(11)	N(7)-Mn(2)-O(3)#1	94.43(12)
C(3)-O(3)-Mn(2)	134.4(3)	C(3)-O(3)-Mn(2)#1	120.1(2)
Mn(2)-O(3)-Mn(2)#1	105.49(12)	C(16)-N(7)-Mn(2)	122.2(3)
C(17)-N(7)-Mn(2)	131.5(3)	C(1)-O(2)-Mn(2)	141.3(3)
O(1)-Mn(1)-O(1)#3	180.00(17)	C(1)-O(1)-Mn(1)	122.5(3)
C(15)-O(12)-Mn(2)#1	104.1(2)		
Symmetry codes: #1 -x+1,-y,-z; #2 -x+2,-y-1,-z+1; #3 -x,-y+1,-z			

Table S3. Complexes synthesized using H_6L ligands in the literature.

Compound	Dimensionality	Coordination number of carboxylic acid	Ref.
Compound **1**	3D	7	Q. L. Zhu, T. L. Sheng, R. B. Fu, S.M. Hu, C.J. Shen, X. Ma, X. T. Wu, CrystEngComm, 2011, 13, 2096.
Compound **2**	3D	5, 8	
Compound **3**	3D	8,8	
Compound **4**	3D	9	
Compound **1**	3D	16	P. K. Goswami, M. Singh, R. Thaimattam and A. Ramanan, CrystEngComm, 2013, 15, 9787.
Compound **2**	3D	8	
Compound **3a**	1D	4	
Compound **3b**	2D	5	
Compound **4a**	2D	8	
Compound **4b**	1D	3	
Compound **1**	3D	8	S. Li, J. Song, J. C. Ni, Z. N. Wang, X. Gao, Z. Shi, F. Y. Bai, Y. H. Xing, RSC Adv., 2016, 6, 36000
Compound **2**	3D	9	

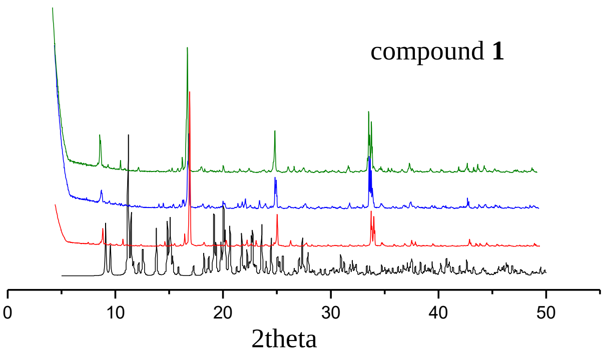

Figure S1. Simulated (black), experimental (red), immersed in water after 2 days (blue) and immersed in Fe^{3+} (0.01M) after 2 day (green) powder X-ray diffraction patterns of compound **1**

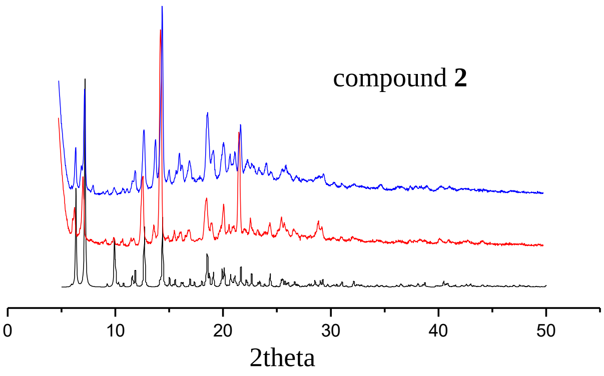

Figure S2. Simulated (black), experimental (red) and immersed in water after 2 days (blue) powder X-ray diffraction patterns of compound **2**

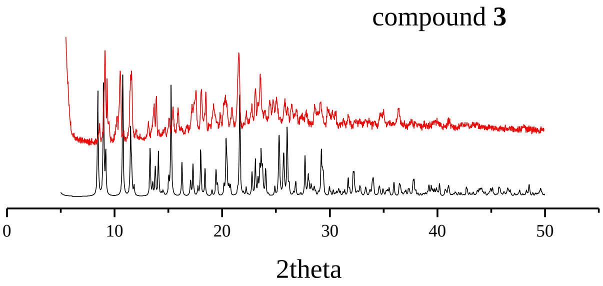

Figure S3. Simulated (black) and experimental (red) powder X-ray diffraction patterns of compound **3**

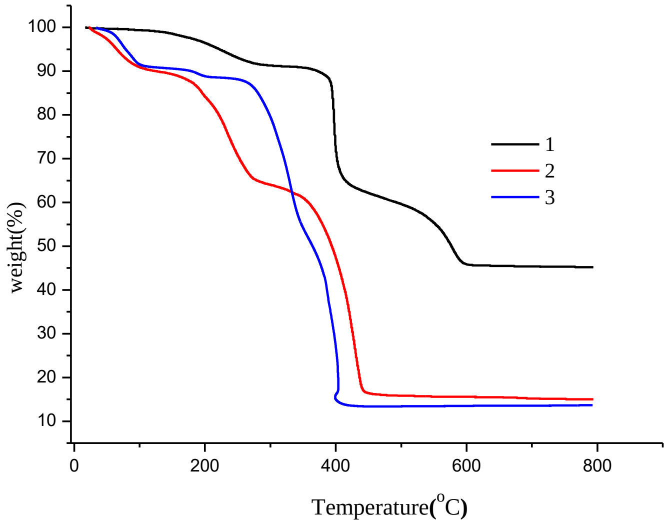

Figure S4. The TGA diagrams of **1**, **2** and **3**

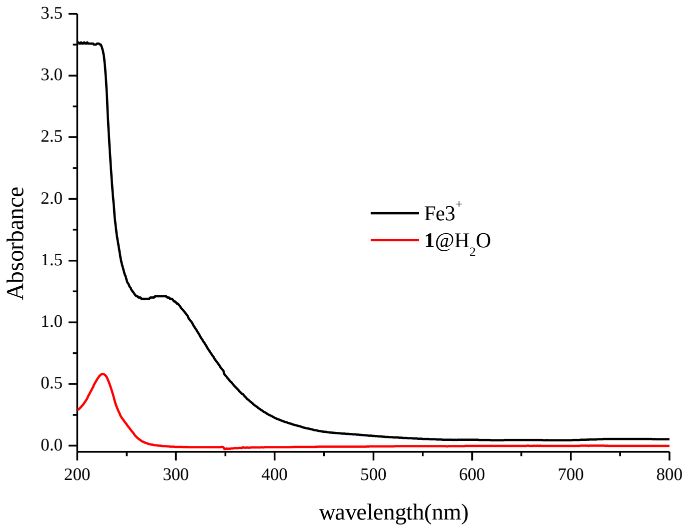

Figure S5. UV-Vis spectra of Fe^{3+}, complex **1** in aqueous solution

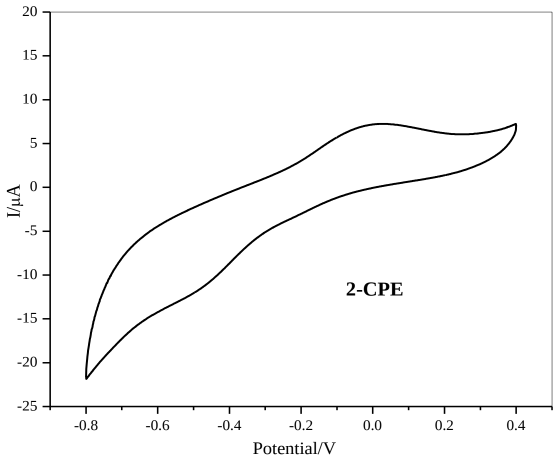

Figure S6. CV of **2-CPE** in 0.5 M NaOH solution at 50 mV/s
